# Locomotion and the early Mesozoic success of Archosauromorpha

**DOI:** 10.1098/rsos.231495

**Published:** 2024-02-07

**Authors:** Amy E. Shipley, Armin Elsler, Suresh A. Singh, Thomas L. Stubbs, Michael J. Benton

**Affiliations:** ^1^ School of Earth Sciences, University of Bristol, Wills Memorial Building, Queen's Road, Bristol BS8 1RJ, UK; ^2^ School of Earth and Environment, University of Leeds, Leeds LS2 9JT, UK; ^3^ School of Life, Health and Chemical Sciences, The Open University, Walton Hall, Milton Keynes MK7 6AA, UK

**Keywords:** diversity, archosauromorphs, archosaurs, avemetatarsalians, dinosaurs, pseudosuchians

## Abstract

The Triassic was a time of ecological upheaval as life recovered from the Permian-Triassic mass extinction. Archosauromorphs were a key component of the recovery, diversifying substantially during the Triassic and encompassing the origins of dinosaurs, pterosaurs and crocodylomorphs. Here, we explore the evolution of locomotion in Archosauromorpha to test whether dinosaurs show any distinctive locomotory features that might explain their success. We implement geometric morphometrics on limb bone shapes and use limb ratios to calculate bipedality and cursoriality metrics. We find that the Avemetatarsalia (dinosaurs, pterosaurs and relatives) exhibit more variable limb form and limb ratios than any other group, indicating a wider range of locomotory modes. The earliest avemetatarsalians were bipedal and cursorial, and their range of form increased through the Triassic with notable diversification shifts following extinction events. This is especially true of dinosaurs, even though these changes cannot be discriminated from a stochastic process. By contrast, the Pseudosuchia (crocodilians and relatives) were more restricted in limb form and locomotor mode with disparity decreasing through time, suggesting more limited locomotor adaptation and vulnerability to extinction. Perhaps the greater locomotor plasticity of dinosaurs gave them a competitive advantage in the changing climates of the Late Triassic.

## 1. Introduction

Dinosaurs dominated terrestrial ecosystems for over 160 million years during the Mesozoic, whether measured in terms of diversity or relative abundance [1–3]. The oldest definite fossils of dinosaurs are known from the late Carnian (230 Ma), but their phylogeny places their origin in the Early Triassic, in the immediate aftermath of the Permian-Triassic mass extinction (PTME), 252 Ma [4–9].

Dinosaurs are members of the clade Avemetatarsalia, which includes pterosaurs as well as two clades that originated in the Middle Triassic (Anisian), the Aphanosauria, quadrupedal forms such as *Teleocrater* and *Dongusuchus*, and the Silesauridae such as *Asilisaurus*. Their sister clade, the Pseudosuchia, originated in the Early Triassic (e.g. the ctenosauriscid *Ctenosauriscus*), confirming that in fact both Pseudosuchia and Avemetatarsalia arose at this unexpectedly early point in the Triassic. Even though Pseudosuchia displayed greater species richness and greater overall morphological diversity than dinosaurs in Triassic ecosystems [10,11], only a few pseudosuchians in the form of crocodylomorphs survived past the Triassic-Jurassic boundary [4,12,13].

Dinosaurs achieved ecological dominance by a three-step process [4,10,14–21]. Beginning with their origin in the Early Triassic (250 Ma), they initially existed at very low diversity and abundance until in the late Carnian (230 Ma) dinosaurs began to diversify following the Carnian Pluvial Event (CPE; 233–232 Ma). Sauropodomorphs and theropods then diversified in the Norian, followed by ornithischians including armoured forms in the Early Jurassic after the end-Triassic mass extinction (ETME; 201.3 Ma). The success and rise to dominance of dinosaurs has often been ascribed to anatomical innovations, especially those related to their locomotion [8,22–28], and some authors explicitly suggested that these locomotory improvements had been actively selected for and gave the dinosaurs competitive advantages (e.g. [22,25,26,28]).

Locomotor changes in tetrapods had already begun in the Late Permian through the transition from a sprawling to an erect posture [29,30]. In a sprawling posture, movement of the limbs causes lateral bending of the vertebral column which compresses the lungs and limits ventilation [31]. An erect posture positions the limbs underneath the body, so this bending does not occur, thus removing any respiratory constraint and allowing increased efficiency during locomotion. The origins of erect postures in archosauromorphs paralleled a similar posture shift in synapsids, perhaps speeded by the EPME, and associated with advancing endothermy in both clades [8]. In both cases, erect stance enabled parasagittal locomotion, where the limbs swing back and forwards in line with the vertebral column, rather than swinging laterally as in a sprawler.

Alongside erect stance came bipedality [32–34] in all avemetatarsalians, and especially in dinosaurs, but a few pseudosuchians became bipedal [35,36]. Bipedality and cursorial ability occur together in the first dinosaurs [34,36–38], enabling them to achieve faster running speeds for short bursts [39]. Numerous aspects of disparity have been compared between early dinosaurs and pseudosuchians [6,16,40,41], with a focus on hind limb bones and posture [42] but not the forelimb bones.

Here, we test the hypothesis that locomotor advances in dinosaurs aided their survival and success in comparison to pseudosuchians in the Triassic and Jurassic. We measure the morphological disparity of key limb bones using geometric morphometrics and calculate the range of limb ratios to assess the roles of bipedality and cursoriality in pseudosuchians and avemetatarsalians through time. Further, we directly test prior hypotheses that locomotor advances were actively selected for among dinosaurs using a quantitative approach relying on evolutionary rate differences [43]. We do not consider all indicators of posture and gait, such as the dorsal and caudal vertebrae of Triassic archosaurs, but focus on the limb elements which are more directly comparable.

## 2. Material and methods

### 2.1. Data sample

We compiled a dataset of all valid species of early Avemetatarsalia, Pseudosuchia and early diverging members of the wider group, the Archosauromorpha, from their Permian origin to the Early Jurassic (259.1–174.1 Ma). We examined literature on all 450 candidate species, of which 208 yielded useful information on limb bones (electronic supplementary material, table S1). The other 242 taxa were excluded because they either lacked any limb bones, the limb bones were damaged or distorted, they pertained exclusively to juveniles, or the literature did not provide adequate illustrations.

For each of the 208 included taxa, we examined images (photographs, specimen drawings) and measurements of key elements of the hindlimb (femur, tibia, fibula, metatarsal III) and forelimb (humerus, radius, ulna, metacarpal III). Images were collected in lateral view of all elements, except metatarsal III, for which dorsal images were most common. In all cases, we included images only of complete, undistorted elements.

The measurements collected were the maximum lengths for each of the elements, taken preferably from numerical data in the text or tables, but otherwise measured by us from scaled images. In such cases, we took length measurements using the image processing software Fiji [44]. Where more than one specimen was recorded for a single species, the largest specimen was used and if necessary other measurements were scaled up to this larger size under the assumption of isometry [45].

### 2.2. Geometric morphometric analysis

A two-dimensional geometric morphometric analysis was performed for each limb bone. As fewer than 10 specimens in one view were recovered for metacarpal III, this bone was not further included. We landmarked each bone using three fixed landmarks and three semi-landmark curves. The first fixed landmark is placed at the proximal end of each limb bone, on top of the proximal head. This landmark is positioned at the most central point, so in some cases (for example taxa with enlarged deltopectoral crests in the humeri or cnemial crests in the tibiae) this does not mean the most proximal point of that bone in lateral view. Two fixed landmarks are further placed on the distal end, one on each of the two processes (or condyles) at their anteriormost and posteriormost points as visible in lateral view. The three semi-landmark curves were then interpolated between these points, two with 18 semi-landmark points each, and one with eight. This landmarking regime is illustrated in the electronic supplementary material.

Landmarks were digitized using the software tpsDig [46] and curves appended in tpsUtil [47] using the chord min *d*^2^ semi-landmark sliding method. A generalized Procrustes analysis was performed in tpsRelW [48] to generate aligned coordinate data by removing the impact of size, position and rotation. Principal component analyses were then carried out in R [49], using the package geomorph [50] to identify the primary axes of variation.

Morphospaces were generated for each bone using the first two principal component axes (PC1, PC2) with convex hulls distinguishing between the taxonomic groups of non-archosaurian archosauromorphs, pseudosuchians, dinosaurs and non-dinosaurian avemetatarsalians (henceforth, ‘other avemetatarsalians'). Dinosaurs were partitioned as a separate group to allow for more direct comparison between these taxa and pseudosuchians to enable us to address previous morphological disparity analyses and ideas of competition between these two groups [6,16,26,28,40,41]. Morphospaces were further split into time bins to analyse change through time.

### 2.3. Statistical tests

Disparity through time was measured using the sum of variances (SoV). This metric was chosen because of its resilience against unequal sample sizes between groups and its ability to better capture changes in the size of occupied trait space than other measures [51,52]. SoV was calculated for the PC values of the femur, tibia and humerus for time bins subdivided at age level. Bins that contained fewer than two species were combined. These calculations were performed in R using the package dispRity [53] with 2000 bootstrap replications.

Significant differences in disparity between the different taxonomic groups and across the CPE and ETME were also assessed through an NPMANOVA multivariate analysis. PC values for all axes were input for the four taxonomic groups and were assessed firstly for differences in disparity across all taxa, and secondly for taxa within time bins (pre-CPE, CPE-ETME and post-ETME). This was performed in the statistical software PAST [54], with 9999 permutations and assessed with Bonferroni correction.

### 2.4. Quadrupedality and cursoriality indices

Limb proportions can be diagnostic of locomotor mode and have often been used to infer the locomotion of extinct taxa [35,39,55]. Here two indices representing quadrupedality and cursoriality are calculated. These indices have been used to assess locomotor modes of archosaurs previously, but for fewer taxa [36].

The quadrupedality index assesses the relative length of the forelimb to the hindlimb, where a shorter forelimb can indicate a more bipedal state [56], and the cursoriality index the length of the metatarsal III to the femur, where a longer metatarsal suggests better adaptation for running [36]:

$$\mathrm{quadrupedality}=\frac{\mathrm{humerus}+\mathrm{radius}}{\mathrm{femur}+\mathrm{tibia}},$$

$$\mathrm{cursoriality}=\frac{\mathrm{metatarsal}\;\mathrm{III}}{\mathrm{femur}}.$$

As with all models, there are limitations to the predictive power of these indices in assigning locomotor mode. Therefore, we are cautious in interpreting specific locomotor modes; taxa are not designated a specific mode, but instead depending on their relative position on the index are assumed to have better adaptation and propensity for one mode over another. This provides adequate interpretation for the questions being addressed in this study. Further, the range occupied by taxa on each index can give an indication of the diversity of locomotor forms exhibited, where a wider range indicates greater disparity and range of modes.

The two metrics can only infer locomotor mode for terrestrial taxa, thus flying and fully aquatic species in the dataset were excluded. These metrics were considered between taxonomic groups and through time.

Quadrupedality and cursoriality are dependent on body size with biomechanical constraints of increased size providing significant limitations [57–59], so body size is also taken into consideration. Body size was inferred from femur length, a metric that has previously been used in other studies on archosaurs [45,60] and is considered a reasonable proxy [38,60]. Femur length is not a perfect estimator of original body mass because isometry in limb bone growth does not always apply [61]. Further, there are alternative estimators of body mass that have greater biomechanical justification and accuracy, such as diaphyseal femur and humerus circumference [2,62], but these measurements are rarely reported, and their impact would be to reduce our dataset drastically, or these values would have had to be estimated which would introduce a further risk of error into the calculations. To assess the relationship between these two indices and body size for Dinosauria and Pseudosuchia, femur length was plotted against these metrics to check for any correlation (electronic supplementary material, figure S4).

Additionally, the cursoriality index of dinosaurs and pseudosuchians was compared using a phylogenetic analysis of covariance (ANCOVA), accounting for the effect of body size. The ANCOVA was run using the cursoriality index as the dependent variable, the two taxonomic groups (Pseudosuchia and Dinosauria) as the grouping variable, and body size (= log_10_-transformed femur length) as the covariate. A random cal3 time-scaled tree (as detailed below) served as input phylogeny. Pagel's *λ* was estimated from the dataset. We implemented the model using the pgls() function of the R package caper [63].

### 2.5. Phylogenetic topology

An informal supertree approach was taken to place all valid archosauromorph species (439 species) in a phylogeny. We used all archosauromorph species as complete trees are preferable over pruned ones when doing the subsequent time-scaling [64,65]. The most comprehensive currently available phylogeny of Archosauromorpha [66] was used as the scaffold for the supertree and additional taxa were added using Mesquite 3.51 [67]. When selecting phylogenies for the supertree construction, preference was given to recent analyses featuring taxon- and character-rich data matrices. We generated two different topologies to reflect the current uncertainty on dinosaur relationships. The first topology represents the consensus on dinosaur relationships [68–74], the second is based on the Ornithoscelida hypothesis [75]. Taxa that have never been included in a phylogenetic analysis were added based on thorough consideration of current taxonomic opinion [76].

### 2.6. Phylogenetic time-scaling

Taxa were dated at geological substage level. Age ranges were based on the most recent information available for each taxon. Absolute ages for geological stages were based on the 2019/05 version of the International Chronostratigraphic Chart [77]. To make sure that our results were not dependent on the chosen time-scaling method, we used three different approaches to time-scale our phylogeny: the R implementations [49] of the cal3 approach [78,79] and the whole tree extended Hedman algorithm [80,81], and the fossilized birth–death (FBD) tip-dating approach [82–84] as implemented in MrBayes v.3.2.7a [85,86]. Polytomies were randomly resolved prior to time-scaling with the cal3 and Hedman approaches generating 100 input trees. We generated a set of 100 time-scaled trees for each time-scaling approach.

The cal3 approach was carried out using the bin_cal3TimePaleoPhy function of the paleotree package [78,79]. The cal3 method is a probabilistic ‘a posteriori’ time-scaling approach [81] which draws divergence times under a birth-death sampling model based on *a priori* known rates of branching, extinction, and sampling [79,86,87]. Calculating sampling rates is a non-trivial exercise [87], especially for terrestrial tetrapods [2]. We therefore decided to obtain the instantaneous sampling rate from a uniform distribution bounded by the lowest (0.018 per lineage million years (lmy^−1^)) and highest estimates (0.18 lmy^−1^) reported in the literature for Devonian tetrapods and Mesozoic dinosaurs [87,88]. These sampling rate estimates were then used to calculate the extinction and origination rates as in Lloyd *et al.* [81]. The time of observation was treated as uncertain and was randomly sampled between the first and last appearance times (dateTreatment: randObs), the step size of increments used in the function to set node ages was set to 0.001 (step.size = 0.001), and the probability of inferring ancestor-descendant relationships was set to 0 (anc.wt = 0). The cal3 algorithm tends to produce several zero-length branches (ZLBs; e.g. [86]) which can be problematic for some phylogenetic comparative methods. To overcome the problem of ZLBs we added 0.0001 Myr (= 100 yr) to all branches with a length of zero (see also [87,89]).

For the Hedman method we used the ‘conservative approach’ [80], which ignores taxa that are younger than the preceding outgroup and set the absolute maximum bound t_0_ conservatively to the base of the Cambrian (542 Ma) following [81]. We used the last appearance dates (LADs) of *Ichthyostega stensioi* (363.3 Ma), *Ymeria denticulata* (358.9 Ma), *Tulerpeton curtum* (358.9 Ma), *Ossirarus kierani* (350.8 Ma), *Casineria kiddi* (336.2 Ma), *Palaeomolgophis scoticus* (336.2 Ma), *Hylonomus lyelli* (315.2 Ma), *Anthracodromeus longipes* (307 Ma), *Petrolacosaurus kansensis* (303.7 Ma), *Orovenator mayorum* (286.8 Ma), *Lanthanolania ivakhnenkoi* (265.1 Ma), and *Eunotosaurus africanus* (259.1 Ma) as outgroup ages. Resolution was set to 10 000. To account for uncertainty in dating we randomly sampled the tip age of each archosauromorph species from a uniform distribution bounded by its first appearance dates (FADs) and LADs.

Topological constraints for the MrBayes FBD tip-dating approach were based on the archosauromorph topology and an ‘empty’ morphological matrix was generated using the createMrBayesTipDatingNexus function of paleotree [78]. Tip age calibrations were defined as uniform priors bounded by the FADs and LADs of the tips. Similar to previous approaches [90–93] we placed a uniform prior on the root of the tree bounded by the FAD of the oldest tip on the tree (*Eorasaurus olsoni*: 259.1 Ma) and a tree age offset of 8.35 Myr (see also Sookias *et al*. [60]). *Vonhuenia friedrichi* was used as the anchor taxon to place the time-scaled trees in absolute time. We used the default FBD and clock priors provided by createMrBayesTipDatingNexus: fossilization rate = beta (1, 1); speciation rate = uniform (0, 10); extinction rate = beta (1, 1); sampling strategy = random; sampling probability = 1; clock rate = normal (0.0025, 0.1); clock variance = igr; igr variance = uniform (0.0001, 200) (following [93]). The analysis was run four times, using six chains per run, for 1 000 000 000 generations and sampling every 4000. Convergence was assessed using Tracer v.1.7.1 [94] with the effective sample size (ESS) of all parameters exceeding 200. The obtainDatedPosteriorTreesMrB function of paleotree was subsequently used to obtain a sample of 100 time-scaled trees from the posterior, employing a burn-in of 20%.

For visualization purposes only, an additional consensus tree including polytomies was time-scaled using the minimum branch length (MBL) method with a minimum branch length of 0.1 myr [95] as implemented in the timePaleoPhy function of the paleotree package.

To account for the potential influence of topology the cal3 approach was also rerun for the Ornithoscelida hypothesis [75]. We dropped *Nyasasaurus parringtoni* from the Ornithoscelida supertree topology prior to time-scaling the trees owing to the uncertain phylogenetic [74,75,96–98] and stratigraphic placement [6,99] of the taxon and its unusual, derived position within the Ornithoscelida topology [75].

### 2.7. Evolutionary rates and test for positive phenotypic selection

We employed phylogenetic comparative methods to obtain evolutionary rates of cursoriality and to detect positive phenotypic selection on cursoriality in early archosauromorphs. We pruned the time-scaled trees a posteriori to include only taxa with cursoriality index. None of the taxa placed according to taxonomic opinion (and which have never been included in phylogenetic analysis before) is part of a polytomy in the pruned trees. Problems related to randomly resolving polytomies [100] therefore do not apply to this dataset. We used BayesTraits v2.0.2 (http://www.evolution.rdg.ac.uk/BayesTraitsV2.0.2.html) [101] to estimate multivariate variable rates models for the cursoriality index. We ran variable rates independent contrast models using the Markov chain Monte Carlo method with default priors for each time-scaled tree. Each tree was run for 1 000 000 000 iterations and parameters were sampled every 80 000 iterations; 200 000 000 iterations were discarded as burn-in. We calculated the marginal likelihood of the models using the stepping stone sampler [102] implemented in BayesTraits. We sampled 1000 stones and used 100,000 iterations per stone. Convergence was assessed using the R package CODA [103], making sure that the smallest ESS value across all 100 trees exceeded greater than 200. We used the variable rates post processor (http://www.evolution.reading.ac.uk/VarRatesWebPP/) [43] to extract the final parameters results. Models were compared using a Bayes factor (BF) test. We calculated a strict consensus tree for all time-scaled trees in which the branch lengths had been replaced with the mean rate scalars calculated by BayesTraits. The consensus tree was computed using the R package phytools [104]. We calculated the mean branch lengths for each set of trees, ignoring edges that were not present in all trees of a set. We then plotted the (rescaled) branch lengths of the consensus tree onto the MBL tree using ggtree [105] with branches colour-coded according to the mean rate scalars.

Using the fastAnc() function of the R package phytools [104] we also estimated the ancestral cursoriality index at internal nodes of the archosauromorph phylogeny. To account for the heterogeneity in evolutionary rates, we calculated a consensus tree for all time-scaled trees in which the branch lengths had been multiplied with the mean rate scalars calculated by BayesTraits. The consensus tree was again computed using phytools [104]. We calculated the resulting mean branch lengths, ignoring edges that were not present in all trees of the chosen topology. This consensus tree then served as input phylogeny for the fastAnc() function to obtain ancestral cursoriality index estimates which account for rate heterogeneity. We then generated a phenogram using ggtree [105] based on the MBL tree, the measured cursoriality indices, the ancestral state estimates, and the femur lengths which served as body size proxy [60]. Branches were colour-coded according to the mean rate scalars.

Positive phenotypic selection *sensu* Baker *et al*. [43] was defined on the basis of two criteria: (i) the ratio between the expected phenotypic variance on a single branch owing to rate variation and the expected phenotypic variance given a Brownian motion background rate is larger than 2 (magnitude criterion); and (ii) this ratio must be observed in more than 95% of the posterior distribution of rate scalars of the respective branch (certainty criterion) [43]. Positive phenotypic selection is therefore detected on a branch if the rate scalar for this branch is greater than 2 in greater than 95% of the posterior distribution of rescaled trees output by BayesTraits. The definition is inspired by the ratio of the non-synonymous rate of nucleotide substitutions to the synonymous rate of nucleotide substitutions in protein-coding genes d*N*/d*S*, where positive selection is detected if the non-synonymous rate of substitution contributes to more than half of the genetic change observed on the branch of interest [43,106,107]. This definition assumes that topology and branch lengths of a single phylogenetic tree are reliable for the studied clade [43]. Since we ran the BayesTraits analyses over a set of trees, we added an additional criterion to detect positive phenotypic selection: (iii) positive phenotypic selection is only detected if the magnitude criterion (i) and the certainty criterion (ii) apply to all trees for the branch of interest. We used the modal rate scalar for the magnitude criterion and the scaling frequency of a branch in the posterior distribution for the certainty criterion, both reported by the variable rates post processor [43]. This approach is expected to give very similar results compared to the original definition (J. Baker 2018, personal communication; C. Venditti 2018, personal communication) and greatly reduces the memory footprint. We repeated all analyses for both topologies and all time-scaling approaches.

Rate results could be dependent on other variables such as body size [43]. We therefore also ran independent contrast regression models using BayesTraits v3.0 [101] with the cursoriality index as dependent variable and body size as independent variable. Akin to the approach of [36] we calculated the geometric mean for the sum of femur length and tibia length for each taxon to represent body size, which was log_10_-transformed prior to the regression analyses. Taxa for which tibia length was not available were dropped from the analyses. Parameter settings, priors, post-processing and subsequent plotting corresponded to the independent contrast models without body size. Analyses were again repeated for both topologies and all time-scaling approaches.

## 3. Results

### 3.1. Morphospace occupation

**Figure 1.**
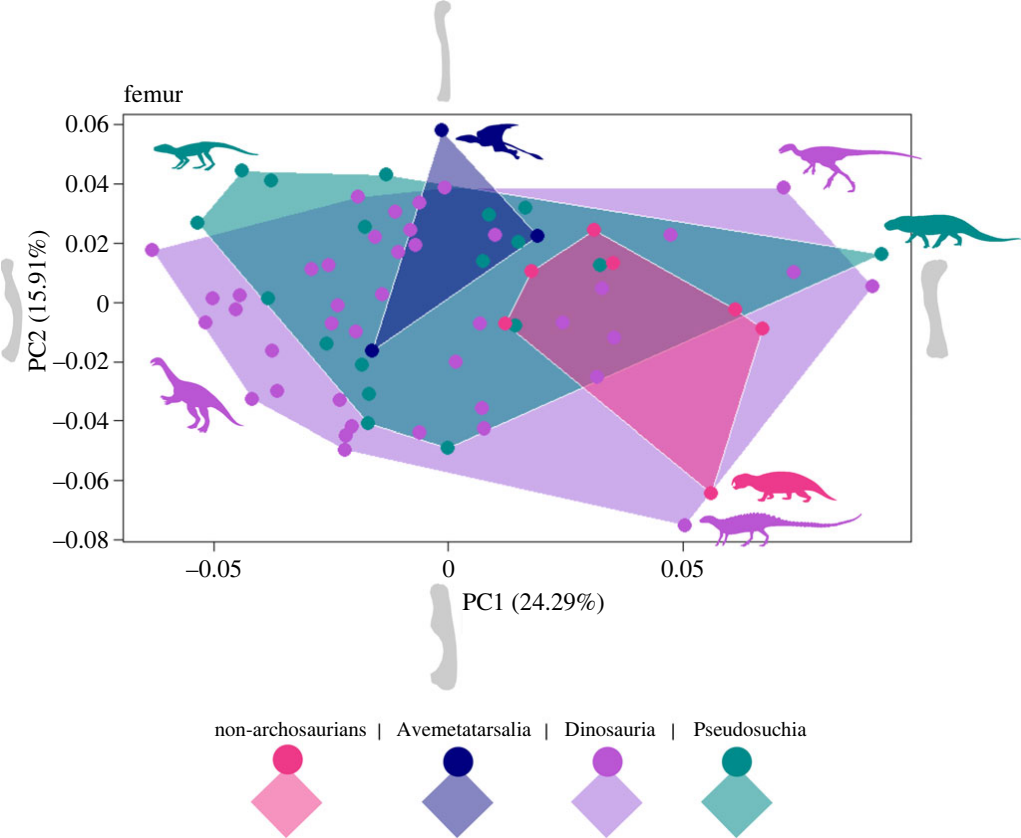
Morphospace occupation for the shape outline of the femur for four groups of archosauromorphs, non-archosaurian archosauromorphs (Rhynchosauria, Prolacertiformes, Trilophosauridae), Avemetatarsalia (excluding Dinosauria), Dinosauria, and Pseudosuchia. Shape of femora at extremes of the PC axes are displayed as silhouettes.

**Figure 2.**
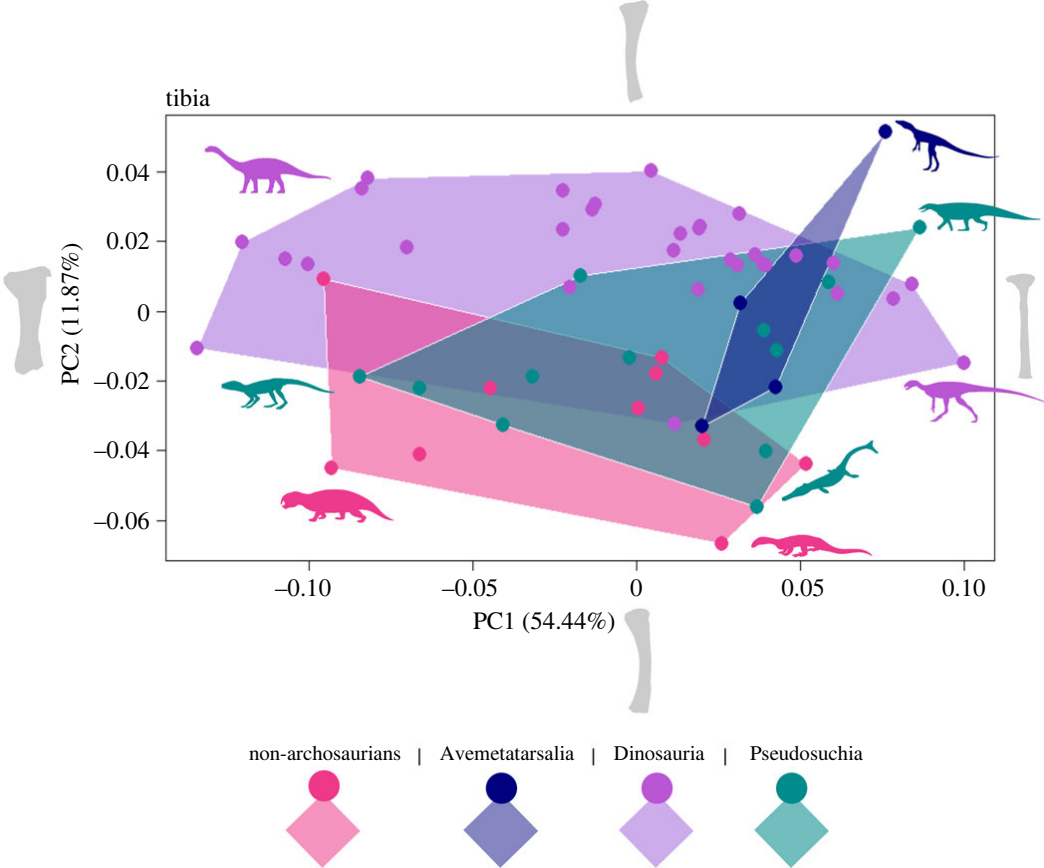
Morphospace occupation for the shape outline of the tibia for four groups of archosauromorphs, non-archosaurian archosauromorphs (Rhynchosauria, Prolacertiformes, Trilophosauridae), Avemetatarsalia (excluding Dinosauria), Dinosauria, and Pseudosuchia. Shape of tibia at extremes of the PC axes are displayed as silhouettes.

**Figure 3.**
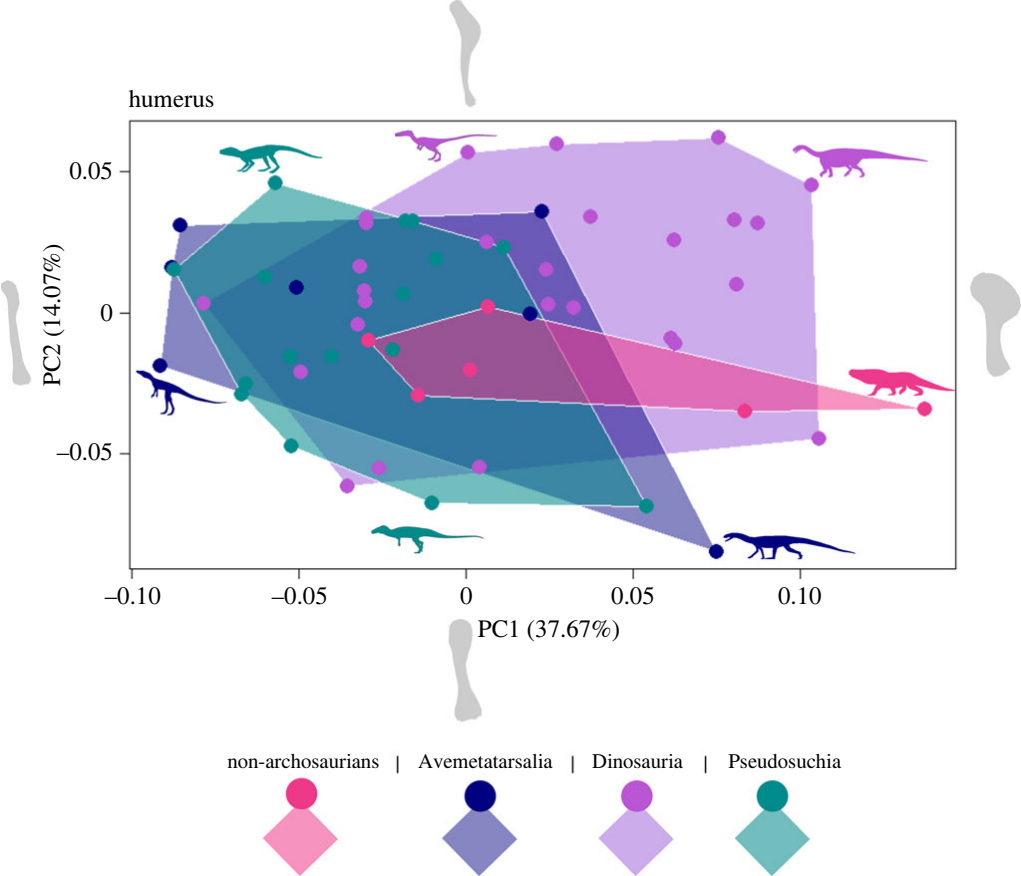
Morphospace occupation for the shape outline of the humerus for four groups of archosauromorphs, non-archosaurian archosauromorphs (Rhynchosauria, Prolacertiformes, Trilophosauridae), Avemetatarsalia (excluding Dinosauria), Dinosauria, and Pseudosuchia. Shape of humeri at extremes of the PC axes are displayed as silhouettes.

Geometric morphometric analyses of the limb bones show considerable similarity in morphospace occupation between the different taxonomic groups for the femur, tibia and humerus (figures 1–3). Results for the other limb elements also show similar overlap of morphospace, but small sample sizes, particularly for metatarsal III, and few non-archosaurian archosauromorph specimens, make these patterns harder to distinguish. Therefore, these are presented in the electronic supplementary material, figure S1.

For the femur, PC1 represents 24.29% of anatomical variation and captures the direction of curvature of the femoral shaft whereas PC2 represents 15.91% of variation, and the relative width and prominence of the fourth trochanter. PC1 of the tibia, (54.44% of variation), instead represents the relative width and expansion of the tibial head, and PC2 (11.87% of variation) the direction of curvature of the tibial shaft. Finally, for the humerus, PC1 (37.67% of variation) captures the expansion of the deltopectoral crest whereas PC2 (14.07%) shows the change in definition of humeral features and the prominence of the distal condyles.

PC axes 3 through to 6 were also assessed to check for any patterns in morphospace occupation not accounted for on PC axes 1 and 2, especially as these axes in the femur represent less than 50% of all shape variation. Although some minor differences were noted (mentioned below), overall morphospace occupation showed very similar relative coverage and overlap of the four taxonomic groups as seen on PC1 and PC2, and thus are presented in the electronic supplementary material, figure S2.

Dinosaurs consistently occupy the largest area of morphospace, in each case spanning a broad region along both PC1 and PC2 (figures 1–3). Species belonging to the other taxonomic groups (non-archosaurian archosauromorphs, non-dinosaurian avemetatarsalians and pseudosuchians) generally lie at the extremes of the dinosaur morphospace, although there are notable outliers for each of the three limb elements.

The femur (figure 1) displays the greatest overlap between dinosaurs and pseudosuchians. The pseudosuchian species nearly all sit within the boundaries of the region occupied by dinosaurs, except those in the area of low PC1 and high PC2 scores (top left). These outliers represent slender femora with high curvature belonging to the similarly sized carnivorous crocodylomorphs *Kayentasuchus walkeria*, *Dromicosuchus grallator* and *Hesperosuchus agilis*. The avemetatarsalian pterosaur *Rhaeticodactylus filisurensis* is also in this region, its delicate femoral form reflecting partly its small size and partly its limb function. There is greater separation between dinosaurs and pseudosuchians in PC3 to PC6 (electronic supplementary material, figure S2*a,b*) with pseudosuchians occupying areas of the morphospace that lack dinosaurs. This is particularly prevalent on PC axes 5 and 6 where pseudosuchians show expansion in the area of high PC5 and low PC6 scores. However, the overall space occupied by the groups on PC axes 3 to 6 is very similar to that seen on PC axes 1 and 2.

The tibia (figure 2) shows the greatest separation between non-archosaurian archosauromorphs and the other groups, extending into the lower area on PC2 with posteriorly curved humeri. Some pseudosuchians also occupy this region, notably the marine thalattosuchian *Magyarosuchus fitosi*. The greatest separation of dinosaurs from other taxonomic groups is seen in the area with low PC1 and high PC2 scores, occupied entirely by sauropodomorphs with wider and more robust tibia. PC axes 3 to 6 however find notably less separation between the non-archosaurian archosauromorphs, overlapping far more with dinosaurs. On PC axes 5 and 6, this group also occupies far less area in the morphospace, and this is also seen in the avemetatarsalians. The separation of dinosaurs from the other groups owing to sauropodomorph taxa is still consistent in these other PC axes.

The humerus (figure 3) shows most overlap between the four groups, with even the avemetatarsalians and non-archosaurian archosauromorphs sharing notable overlap, whereas in the femur these two groups show total separation. Avemetatarsalians also occupy a relatively large area of morphospace unlike for the femur and tibia. Among dinosaurs, the sauropodomorphs occupy their own region of morphospace, even more so than for the tibia. These species have higher PC1 scores which correspond to humeri of greater width and an expanded deltopectoral crest and enlarged humeral head. Only two non-archosaurian archosauromorphs also occupy this area of morphospace on PC axes 1 and 2 (*Hyperodapedon tikiensis*, *Erythrosuchus africanus*), despite their considerable differences in general anatomy and adaptation, but both sharing similar limb proportions and being obligate quadrupeds like many sauropodomorphs. A small number of pseudosuchians and avemetatarsalians extend beyond the dinosaur morphospace region, including the dinosauromorph *Silesaurus opolensis* and crocodylomorph *Terrestrisuchus gracilis* in the lowest areas of PC1 and the aphanosaurian *Yarasuchus deccanensis* and *Prestosuchus chiniquensis* in the lowest areas of PC2. PC axes 3 to 6 exhibit very similar patterns of morphospace occupation to those seen on PC axes 1 and 2. The expansion of sauropodomorphs into their own region of morphospace is evident on PC axes 3 and 4, but this is less clear on PC axes 5 and 6, because these axes place less emphasis on variations in the shape of the deltopectoral crest. Further, there is slightly less overlap between dinosaurs and the other taxonomic groups on PC axes 3 and 4.

**Figure 4.**
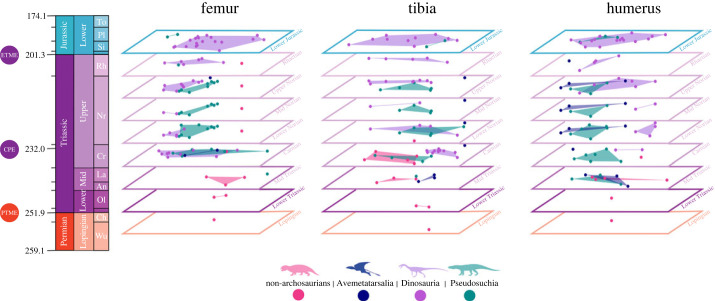
Change in morphospace occupation for each limb element (femur, tibia and humerus) and for each taxonomic group (non-archosaurian archosauromorphs, Avemetatarsalia, Dinosauria and Pseudosuchia) through the Late Permian to Early Jurassic interval. The Permian-Triassic mass extinction (PTME), Carnian Pluvial Event (CPE) and end-Triassic mass extinction (ETME) are indicated on the time-scale. Time slices are presented in standard orientation in the electronic supplementary material.

Changes in morphospace occupation through time (figure 4), including overlaps in morphospace, are not consistent and vary greatly across time bins and between limb bones. There is a general expansion of occupied morphospace through time for all three limb bones, particularly towards higher values on PC1 for the femur and humerus. During the Triassic, dinosaurs and pseudosuchians mostly occupy separate areas of morphospace. The greatest overlaps occur in the femur during the Carnian and the tibia during the early Norian.

The Early Jurassic sees the broadest area of morphospace occupied by the dinosaurs, including regions previously inhabited by the other groups during the Triassic. Inferences for morphospace overlap during the Jurassic are difficult because the sample size for pseudosuchians is small, but at least in terms of humeral shape pseudosuchians do not appear to have differed much from contemporaneous dinosaurs. The femora of the two Early Jurassic pseudosuchians in the sample do however appear different from dinosaurs: these are the terrestrial crocodylomorph *Kayentasuchus walkeri* and the marine metriorhynchoid *Magyarosuchus fitosi*, the latter exhibiting a mode of life never adopted by any dinosaur.

### 3.2. Sum of variances analysis

**Figure 5.**
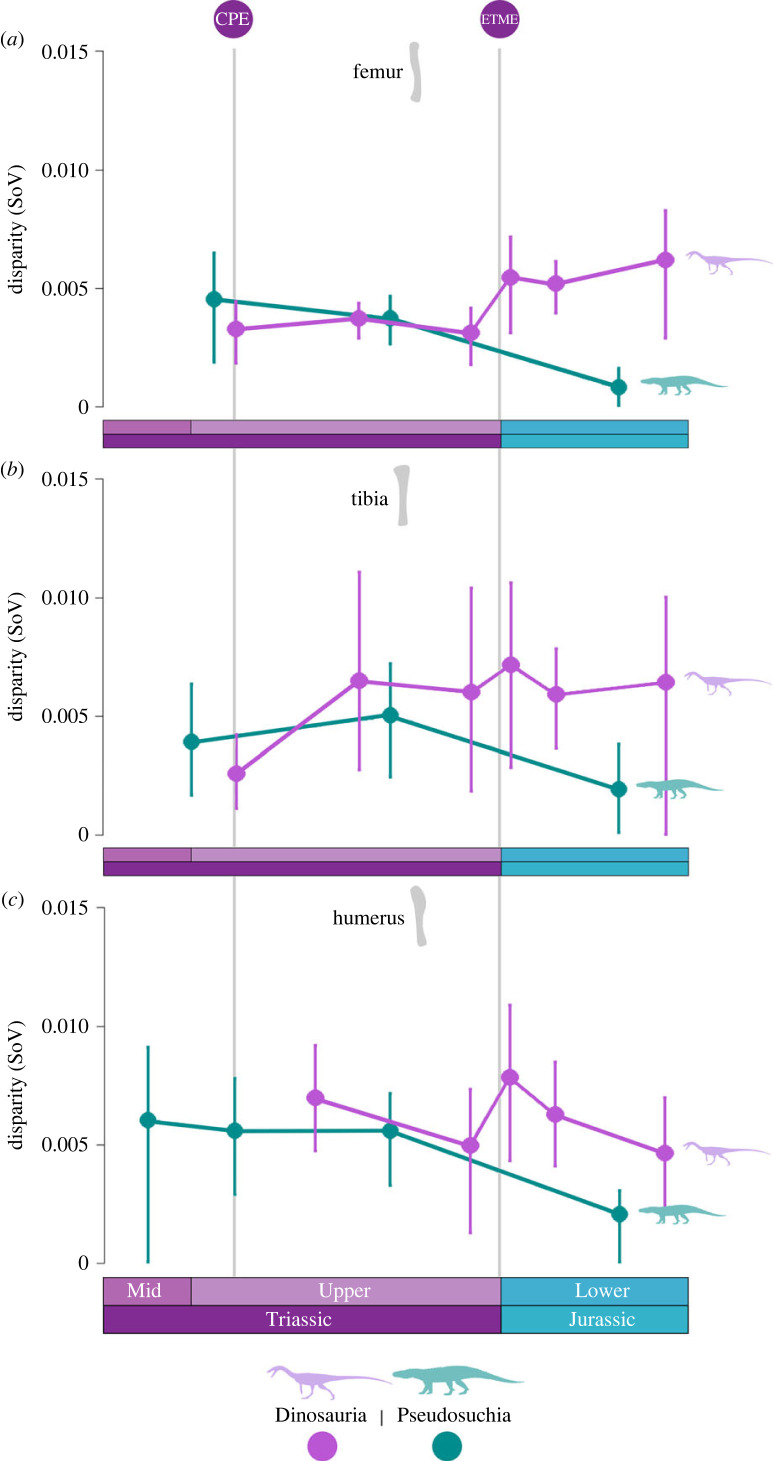
Disparity of limb bone shape for Dinosauria (purple) and Pseudosuchia (blue) measured as sum of variances (SoV) for femur (*a*), tibia (*b*), and humerus (*c*). Owing to small sample sizes, the timespan is Middle Triassic to Early Jurassic, and time bins are large. The Carnian Pluvial Event (CPE) and end-Triassic mass extinction (ETME) are indicated.

SoV trends in disparity (figure 5) show overall higher levels of disparity in dinosaurs than pseudosuchians. However, sample sizes for pseudosuchians were smaller than for dinosaurs and coarser time bins had to be used to allow for a meaningful comparison. Disparity falls continuously through time for Pseudosuchia in all three limb elements, with the greatest drop over the ETME boundary, but detail is lacking because of the use of coarser time bins.

Dinosaurs show much more variation in limb bone shape, indicated by wider error bars, although there is a general trend of increasing disparity over time. Immediately following the CPE, dinosaurs display slightly lower values of disparity than pseudosuchians in the femur and tibia, and slightly higher values in the humerus. However, towards the end of the Triassic and into the Jurassic dinosaurs display distinctly higher values of disparity than pseudosuchians, mainly matching the fall in pseudosuchian disparity. Following the ETME, there is a sharp increase in dinosaur disparity for all three elements, followed by a slight dip and then a subsequent rise in the femur and tibia.

### 3.3. NPMANOVA results

The NPMANOVA tests indicate a significant difference in morphospace occupation between the taxonomic groups (electronic supplementary material, tables S1–S14). Even though dinosaurs occupy the largest area of morphospace for all three limb elements, this was only significantly greater than that occupied by pseudosuchians for the humerus (*p*_bonf_ = 0.0042). There was also a significant difference in femur morphospace occupation between dinosaurs and non-archosaurian archosauromorphs (*p*_bonf_ = 0.003), and pseudosuchians and non-archosaurian archosauromorphs (*p*_bonf_ = 0.0168).

NPMANOVA tests across the Triassic-Jurassic boundary show a significant increase in dinosaur morphospace occupation for the femur (*p*_bonf_ = 0.0048). Although there is a considerable decrease in morphospace area occupied by pseudosuchians following the ETME, the fall is not significant probably because of the small Jurassic sample size. There was no other significant change in any of the groups across this boundary or across the CPE.

### 3.4. Locomotor modes

**Figure 6.**
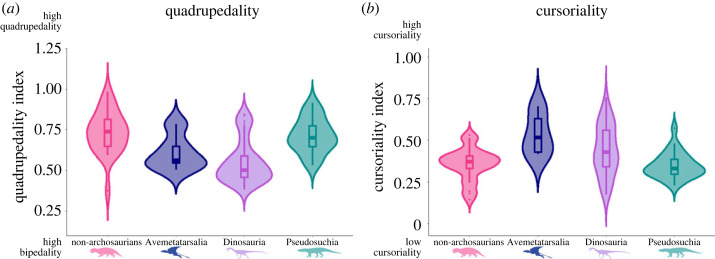
Distribution of quadrupedality index (*a*) and cursoriality index (*b*) for the four taxonomic groups, shown as violin plots of the whole samples for each group. Flying and fully aquatic taxa were removed from these analyses.

The quadrupedality and cursoriality indices vary among the terrestrial taxa (figure 6). There is indeed substantial overlap between the clades, indicating that many species shared similar locomotor modes and forms, similarly to the geometric morphometric data. However, the ranges and modal values for each group differ. It should be noted that these indices are reported as continuous measurements, so discrete forms can only be inferred.

The non-archosaurian archosauromorphs occupy the largest range on the quadrupedality index, having good representation of both bipedal and quadrupedal species. However, the modal value for dinosaurs is lower than any other group, indicating a greater number of species that were probably bipedal. By contrast, most pseudosuchian species have higher values, indicating a greater propensity towards quadrupedality.

Dinosaurs exhibit the greatest spread on the cursoriality index, and while avemetatarsalians have the highest modal value, dinosaurs are on average the next most cursorial group. While several species of non-archosaurian archosauromorphs exhibit lower cursoriality index values, on average pseudosuchians have the lowest values, indicating lower propensity towards cursoriality.

**Figure 7.**
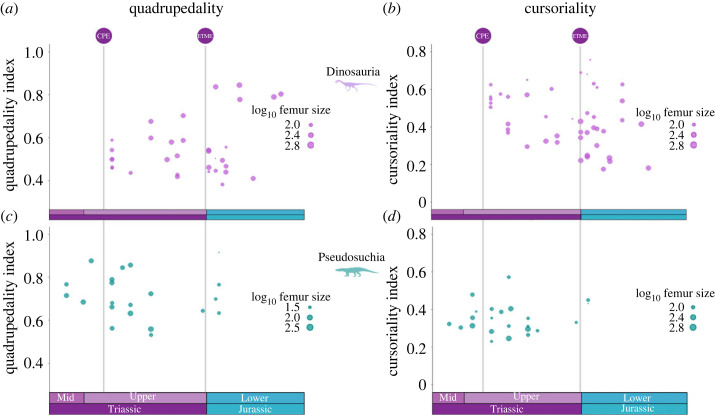
Distribution of quadrupedality index (*a,c*) and cursoriality index (*b,d*) for Dinosauria (*a,b*) and Pseudosuchia (*c,d*) across time. Each point represents measurements for a single species, with the spot size scaled according to the log_10_ femur size as a proxy for overall body size. Flying and fully aquatic taxa were removed from these analyses. The Carnian Pluvial Event (CPE) and end-Triassic mass extinction (ETME) are indicated.

These indices were then considered across time for dinosaurs and pseudosuchians (figure 7). While dinosaur species with a more bipedal gait and more cursorial locomotion occurred right after the CPE, the range occupied by species on both locomotor indices increases through time and by the Jurassic a broad spectrum of forms is seen. Furthermore, across the ETME boundary there appears to be a shift with an increase in spread of approximately 0.18 in quadrupedality and of approximately 0.23 in cursoriality. This expansion in range depends partly on large Jurassic sauropods, occupying the higher end of the quadrupedality index and lower end of the cursoriality index.

By contrast, Pseudosuchia show no apparent change in locomotor indices. Through the Triassic and Jurassic, these taxa remained overall more quadrupedal with a lower propensity for cursoriality than dinosaurs. While there might be a slight increase in range of quadrupedality, including an increase across the CPE of approximately 0.02, this shift is not as pronounced as among dinosaurs. Similarly, pseudosuchian species occupy a narrow range of quadrupedality through time, with little variation.

Despite association between biomechanical limitations of body size and the ability to adopt different locomotor modes, there is no clear relationship between femur length and locomotor mode among Pseudosuchia (electronic supplementary material, figure S4). However, dinosaurs do show some correlation, particularly in the cursoriality index where high values are more associated with smaller body sizes (femur lengths). The quadrupedality index also sees a trend of increased femur length with a greater propensity towards a quadrupedal locomotor mode, as expected, but this relationship is less clear.

The results of the phylogenetic ANCOVA found that the cursoriality indices of dinosaurs were highly significantly different from those of pseudosuchians (electronic supplementary material, table S15), with the Dinosauria exhibiting a larger cursoriality index. Further, there is a significant relationship between this index and body size (log_10_-transformed femur length), where an increase in body size implies a decrease in the cursoriality index and thus an inferred less-cursorial lifestyle.

### 3.5. Evolutionary rates

The heterogeneous rate model is supported across different topologies and time-scaling methods. We find positive evidence for variable rates for the cal3 time-scaled standard topology (log (BF) ≥ 2) in 87 out of 100 trees, strong evidence (log (BF) ≥ 5) in 72 out of 100 trees, and very strong evidence (log(BF) ≥ 10) in 45 out of 100 trees (Hedman: 79, 35, 9; FBD: 92, 84, 69). For the cal3 time-scaled Ornithoscelida topology, the figures are 89, 72 and 37 out of 100 trees, respectively. Figures are similar but slightly lower for rate analyses accounting for body size (cal3: 73, 51, 28; Hedman: 79, 42, 14; FBD: 90, 77, 63; cal3 Ornithoscelida: 68, 49, 21).

**Figure 8.**
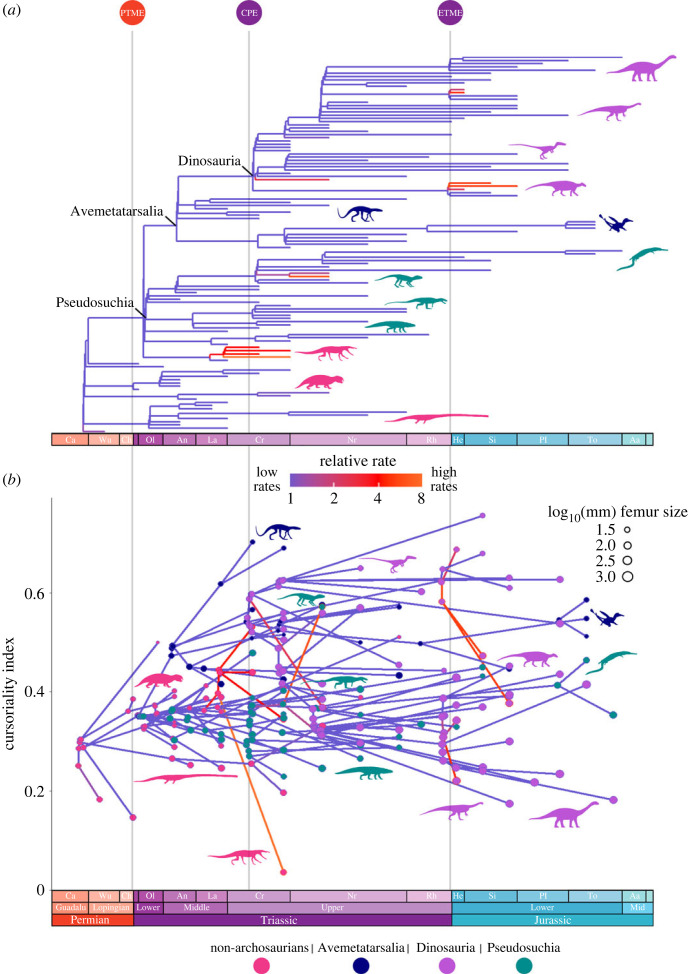
Rates of cursorial evolution of early archosauromorphs based on cal3 time-scaled trees, with four major taxonomic groups indicated by colour: non-archosaurian archosauromorphs (red), Avemetatarsalia (excluding Dinosauria, dark blue), Dinosauria (purple), and Pseudosuchia (green). (*a*) Phylogeny plotted against time, and highlighting evolutionary rate from slow (blue) to fast (red, orange). (*b*) Cursoriality phenogram with branches colour-coded according to evolutionary rates. Body size of external and (reconstructed) internal nodes indicated by circle areas. The Permian-Triassic mass extinction (PTME), Carnian Pluvial Event (CPE) and end-Triassic mass extinction (ETME) are indicated on the timescale.

For all three time-scaling methods, cal3 (figure 8*a*), Hedman (electronic supplementary material, figure S7) and FBD (electronic supplementary material, figure S8), dinosaurs do not show much higher evolutionary rates than the other groups. There is no evidence for phenotypic selection for cursoriality in dinosaurs or any other group, with most changes best explained by the Brownian motion model. Accounting for body size as a potentially confounding factor [43] produces qualitatively similar results (electronic supplementary material, figures S9–S11).

The few instances of high rates in Dinosauria are the early dinosaur *Guaibasaurus candelariensis*, the three ornithischians *Lesothosaurus diagnosticus*, *Scelidosaurus harrisoni* and *Eocursor parvus*, and the relatively large Jurassic sauropodomorphs *Xingxuilong chengi* and *Jingshanosaurus xinwaensis*. Among Pseudosuchia, higher rates are seen in the small and probably agile crocodylomorphs *Hesperosuchus agilis* and *Dromicosuchus grallator*, although only marginally higher in the latter. All representatives of Proterochampsia, except *Jaxtasuchus salomon*, among the non-archosaurian archosauromorphs have relatively high rates, with *Proterochampsa barrionuevoi* presenting the highest rate across the entire phylogeny. No exceptional rates were found in the non-dinosaurian avemetatarsalians.

When these rates are put in context with the cursoriality index (figure 8*b*), those species that exhibit higher than background rates show shifts to both the higher and lower ends of the index. For *Proterochampsa barrionuevoi* this is a substantial drop in cursoriality compared to its closest relatives, and it is the least cursorial of all species in this analysis. Similarly, the ornithischians *Scelidosaurus harrisoni* and *Lesothosaurus diagnosticus* also show drops in cursoriality, whereas *Eocursor parvus* is more cursorial than its relatives. *Hesperosuchus agilis* shows the greatest evolutionary rates, and thus change in cursoriality among the pseudosuchians, being the most cursorial species of this group. Again, there is no obvious trend between body size, cursoriality or phylogenetic position.

## 4. Discussion

The Archosauromorpha diversified significantly into numerous environments and modes of life following the PTME [41,60,108]. This is reflected in the locomotor data presented here, where a great variety of both limb form and modes of locomotion are found not only in dinosaurs and pseudosuchians, but also in the non-dinosaurian avemetatarsalians and the non-archosaurian members of the Archosauromorpha.

The limb bones of these groups all share large areas of morphospace, indicating possibly shared modes of life. However, the overlaps of limb form rarely occur at the same time. This is particularly notable between dinosaurs and pseudosuchians where, although some overlap is seen during the Carnian and Norian, they mostly occupied different areas of morphospace at any time. This suggests divergence in locomotor adaptations between these two groups during the Triassic and that they were not ‘competing’ morphologically. Further, while dinosaurs shared the largest overall area of morphospace relative to the other groups, this is not consistent across time. It was only in the Early Jurassic, when dinosaurs had become the widespread ecologically dominating group, that their morphospace shows the greatest extent, expanding into areas that were occupied by other clades in the Triassic. Several examples of apparent convergence in limb form have been well documented [40], including Late Triassic pseudosuchian poposaurids and rauisuchids with apex dinosaurian carnivores from the Jurassic [109], and Late Triassic pseudosuchian shuvosaurids with Jurassic ornithomimid dinosaurs [110].

It is perhaps unsurprising that dinosaurs exhibit the greatest overall limb disparity during the Triassic and Early Jurassic, matching their great success in terms of faunal abundance and species richness [14,16,17,60]. However, dinosaurs also display greater disparity than pseudosuchians in the Late Triassic even when the pseudosuchians were more species rich and dominant in ecosystems. These findings are contrary to previous disparity studies using various skeletal characters which found that pseudosuchians were significantly more disparate than dinosaurs during the Late Triassic [4,10,16]. The decline in pseudosuchian disparity following the ETME, as demonstrated here, has been found in all studies, including those based on cranial and mandibular elements [12,41,111]. Therefore, while pseudosuchians may have been overall more disparate in the Late Triassic, reflecting their greater abundance and diversity, at least in their limbs they were not as disparate as the less diverse dinosaurs and perhaps did not use so many specialized locomotor modes [6].

This greater range in limb form seen in dinosaurs compared to pseudosuchians is also reflected in the range of locomotor modes. Dinosaurs not only exhibit a wider range of both quadrupedality and cursoriality indices, but this range increased over time, reflecting their radiation and expansion throughout the Late Triassic and Early Jurassic. By contrast, the range of locomotor modes exhibited by pseudosuchians showed little change, particularly for cursoriality where the range was limited through the entire period.

Through the Triassic, both Pseudosuchia and Avemetatarsalia showed evolution from sprawling to erect stances and parasagittal gaits, but the degree to which they were able to change may have differed. The advantages of erect over sprawling stance, especially at higher body masses are well understood; erect stance reduces bending stresses on the limb bones when compared to a sprawling stance [30]. Erect stance therefore permits larger body masses than sprawling stance. Among extant sprawling lizards weight stresses are mitigated by varying stride length and femur rotation which also might have been mechanisms used by stem archosaurs [112]. However, this in turn reduces locomotor efficiency and does not allow for significant increases in size, as with an erect posture. However, although the sprawling-to-erect stance transition happened in both archosaurian clades, Pseudosuchia retained their ‘crurotarsal’ ankle morphology which allows substantial rotation of the lower leg, necessary in sprawling postures [113,114]. The lack of rotation in the dinosaurian ‘mesotarsal’ ankle perhaps provided greater stability necessary for bipedality and in turn a more cursorial lifestyle.

Among the Triassic archosauromorphs, dinosaurs exhibit the greatest propensity for cursoriality, which perhaps gave them some advantages in terms of speed. Several pseudosuchian groups moved towards the more cursorial end of the index during the Triassic, but none reached the higher values of the index achieved by some early dinosaurs. However, it was not all about high levels of cursoriality: many dinosaurs were not cursorial.

The success of dinosaurs has been explained by the suggestion that they were endothermic and the pseudosuchians were not [22,23], and this might have been enhanced by the likelihood that dinosaurs and pterosaurs in the Triassic had some form of insulating feathers [8,115,116]. However, there is evidence that both archosaur lineages exhibited endothermy through much of the Triassic, based on their bone histology, inferred red blood cell size, bone pneumaticity, and other characteristics [8,117–120]. The difference in their longer-term success might be that the avemetatarsalians had more bird-like metabolisms than the pseudosuchians. The presumed avian-type lung of dinosaurs allowed for increased breathing efficiency by separating gas exchange and ventilation, whereas crocodylomorphs showed a reversion from endothermy to ectothermy and non-avian-like lungs, at least from the Early Jurassic onwards [8,118]. Locomotory modes and metabolic rates are linked to growth rates, which were fast in the small crocodylomorph *Terrestrisuchus gracilis* and the erythrosuchids (stem archosaurs), but perhaps slower in other pseudosuchians [60]. This combination of high metabolic rates, fast growth rates and more efficient breathing probably contributed to the exceptionally large sizes achieved by sauropods [121], a form never realised by the Pseudosuchia.

The idea that dinosaurs prevailed over other tetrapods in the Triassic has long been attributed to their locomotor advances [26,28]. As previously found [36], dinosaurs in general were more bipedal and cursorial than pseudosuchians. However, we find it was only after the CPE that dinosaurs were probably solely bipedal and cursorial, and by the end of the Triassic they exhibited a wide variety of locomotory modes, including propensity for slow quadrupedality. Therefore, these results indicate the impact of bipedality and cursoriality on the initial ecological diversification of dinosaurs in the Carnian, but by the end of the Triassic, bipedality and cursoriality were probably not essential for the success of all dinosaurs [16,99]. Instead, perhaps it was the diversity of locomotory modes following the Carnian that played a greater role in this success.

This is corroborated by our evolutionary rates analysis (figure 8), in which dinosaurs did not show higher rates than other groups. These results suggest that cursoriality was not a trait under strong selective pressure and thus contrary to prior hypotheses [26,28], may not have provided a survival or competitive advantage. Further, dinosaurs probably did not radiate as rapidly into new forms following extinction events as suggested, and evolution was not elevated beyond background rates, as seen also for body size trends [60]. This suggests that while great diversity in limb form and function may have aided their survival and rise to dominance, these innovations were not actively selected for and that this process was more stochastic than driven.

Although there is not enough data across the CPE boundary to identify major changes among dinosaurs and other avemetatarsalians, our results do identify notable shifts across the ETME boundary in both limb disparity and range of locomotor modes, supporting the multi-stage pattern of the rise of dinosaurs [4,10,14–21]. This marks an opportunistic expansion by dinosaurs into ecospaces left open following the extinction of nearly all pseudosuchians [14,16,60]. The lack of morphospace overlap between pseudosuchians and dinosaurs suggests that these two groups did not compete, at least in terms of their locomotory capabilities, contrary to some previous hypotheses [22,26]. While these increases in locomotor disparity among dinosaurs following the ETME could indicate non-competitive replacement of pseudosuchians (with dinosaurs only expanding into broader locomotor niches after major drops in pseudosuchian diversity), dinosaur limb disparity was greater than the latter prior to this major extinction event. Further, the locomotor mode range of dinosaurs was wider than for pseudosuchians in the Late Triassic. This suggests that while some non-competitive replacement may have occurred following the ETME, the wider locomotor capabilities of dinosaurs probably aided their success and survival over this event.

## 5. Conclusion

An erect stance and parasagittal gait, permitting faster and more efficient locomotion, has long been thought to have aided dinosaurs in their rise to dominance during the Triassic and Jurassic. Our study suggests that bipedality or cursoriality were not key and that these locomotor advances were not actively selected for. Instead, our results suggest a more stochastic pattern, with the rise of dinosaurs instead potentially attributed to their ability to exhibit greater plasticity in their locomotion. By adopting a wider variety of limb forms and modes, dinosaurs may have been able to occupy more terrestrial habitats and diversify greatly into these following extinction events, particularly the ETME. By contrast, the dominant Triassic pseudosuchians seemingly did not have this flexibility of form and this probably contributed to their downfall in the Jurassic. Our study shows how limb form can indicate key locomotory modes and to test whether differing postures and gaits might have been responsible for the waxing and waning of clades.

## Data Availability

The data and R codes are provided in the electronic supplementary material [122].
